# Supplementary figures and images for: Prostate Cancer’s Silent Partners: Fibroblasts and Their Influence on Glutamine Metabolism Manipulation
Source: Int J Mol Sci. 2024 Aug 27;25(17):9275. doi: 10.3390/ijms25179275 (PMC11394735; doi:10.3390/ijms25179275)

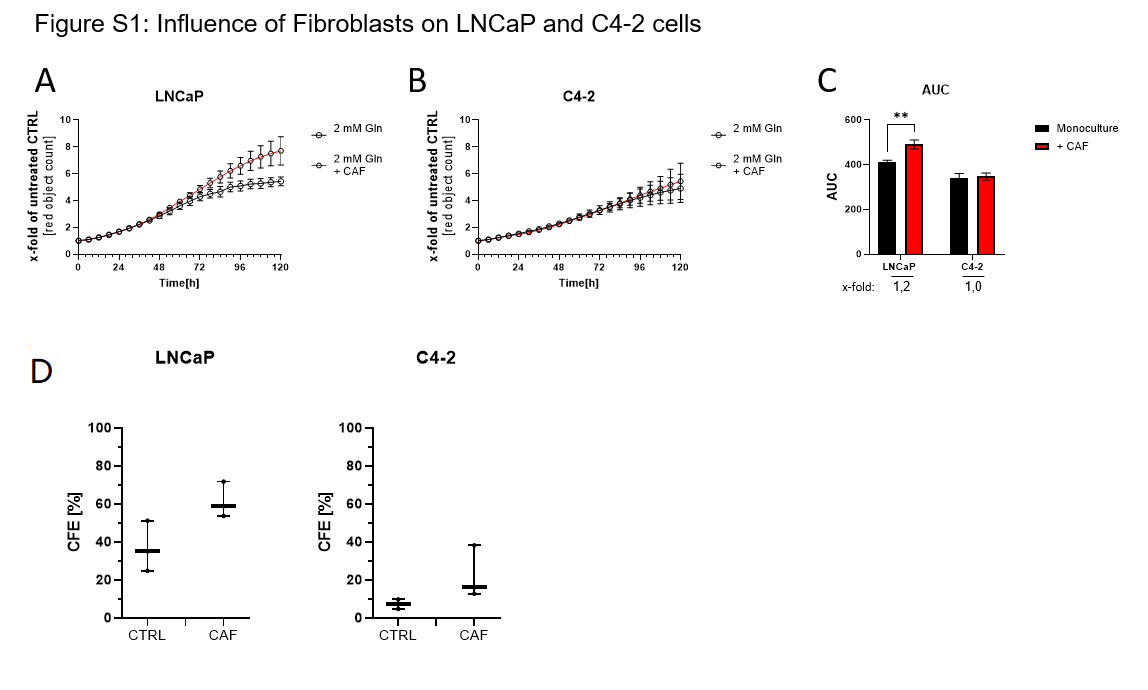

Supplement: Supplementary file 1 [file ijms-25-09275-s001.zip › 20240803_figureS1.tif]

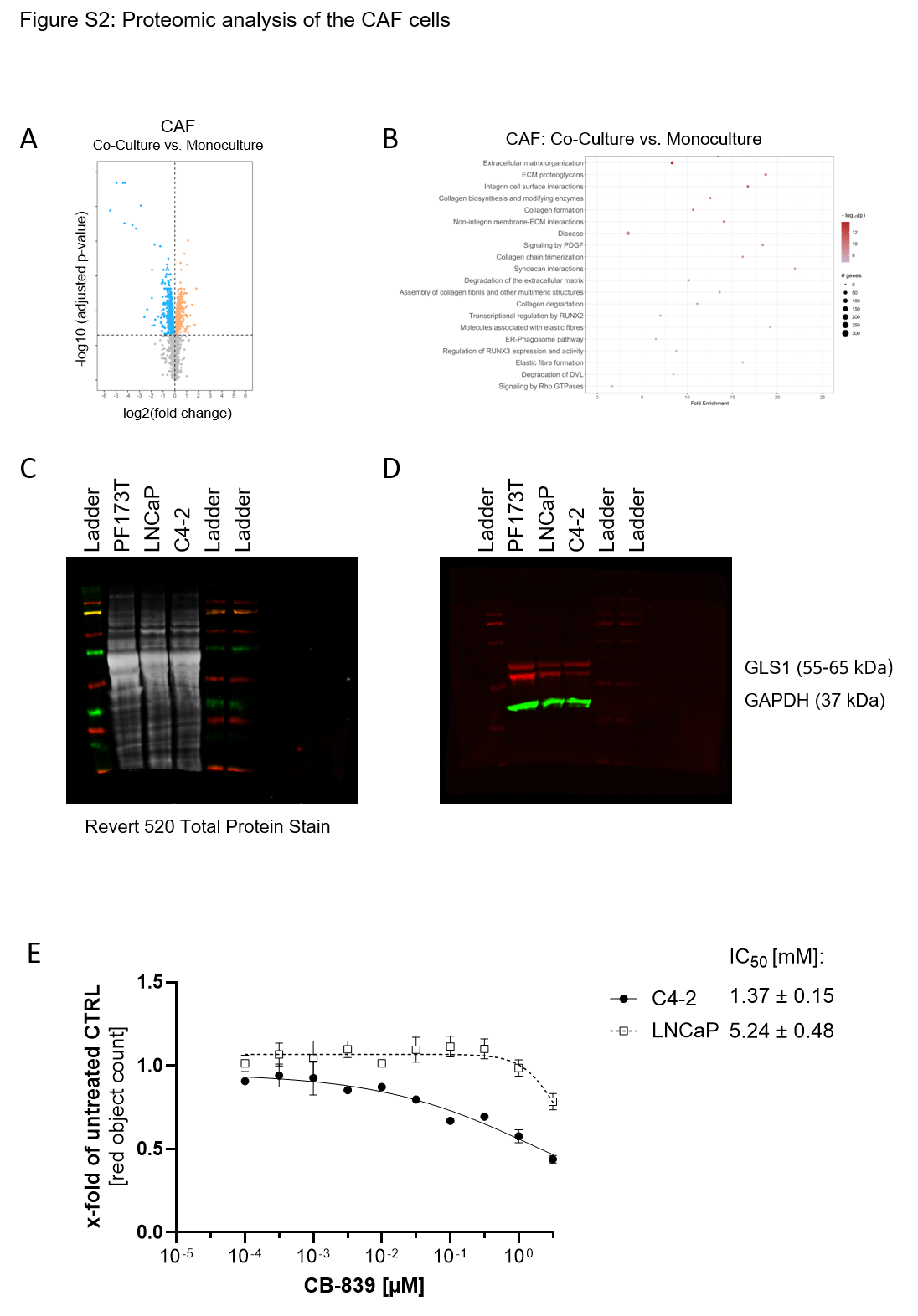

Supplement: Supplementary file 1 [file ijms-25-09275-s001.zip › 20240821_figure_S2.tif]
